# Application of the modified Zajicek criteria to diagnose probable spinal cord neurosarcoidosis

**DOI:** 10.1002/ccr3.1712

**Published:** 2018-07-10

**Authors:** Ceris Ifan Owen, Farrah Jabeen, Anupam Bhattacharjee

**Affiliations:** ^1^ Royal Free Hospital London UK; ^2^ Department of Radiology Royal Free Hospital London UK; ^3^ Department of Neurology Royal Free Hospital London UK

**Keywords:** acute medicine, neurology, respiratory medicine, sarcoidosis

## Abstract

Neurosarcoidosis represents a significant diagnostic challenge, as clinical features overlap with other neuroinflammatory conditions, and biopsy of affected neuronal tissue is often high risk or not feasible. Here we highlight application of the modified Zajicek criteria to diagnose probable spinal neurosarcoidosis in the absence of histology from affected neuronal tissue.

## 1 INTRODUCTION

Sarcoidosis is an idiopathic multisystem disorder characterized by the development of noncaseating granulomas. Symptomatic neurosarcoidosis affects around 5% of sarcoidosis sufferers.^1^ Spinal cord involvement is reported to affect between 10% and 25% of neurosarcoidosis patient and is associated with risk of significant neurological sequelae.^2^ Diagnosis of neurosarcoidosis can be challenging, as this condition may mimic other inflammatory, infective or neoplastic disorders. The individual presented here was diagnosed with probable spinal neurosarcoidosis using the modified Zajicek criteria^3^, ^4^, ^5^ on the basis of characteristic signs of central nervous system (CNS) inflammation on magnetic resonance imaging (MRI) and cerebrospinal fluid (CSF) analysis, coupled with positive histology from a systemic lesion, and a positive high‐resolution CT chest and ^18^F‐FDG PET scan.

## 2 CASE REPORT

A 40‐year‐old man presented with a four‐month history of progressive lower limb weakness and sensory disturbance. He reported occasional fecal incontinence, with associated 8 kg weight loss, and occasional night sweats. He had a past history type 2 diabetes mellitus, but was otherwise well.

Examination revealed proximal lower limb weakness, with a sensory level to L1. Lower limb reflexes were brisk, with flexor plantars. Anal tone and perineal sensation were preserved. The remainder of the neurological examination was unremarkable. Eye examination was normal. General examination was normal with no palpable lymph nodes or skin rashes.

**Figure 1:**
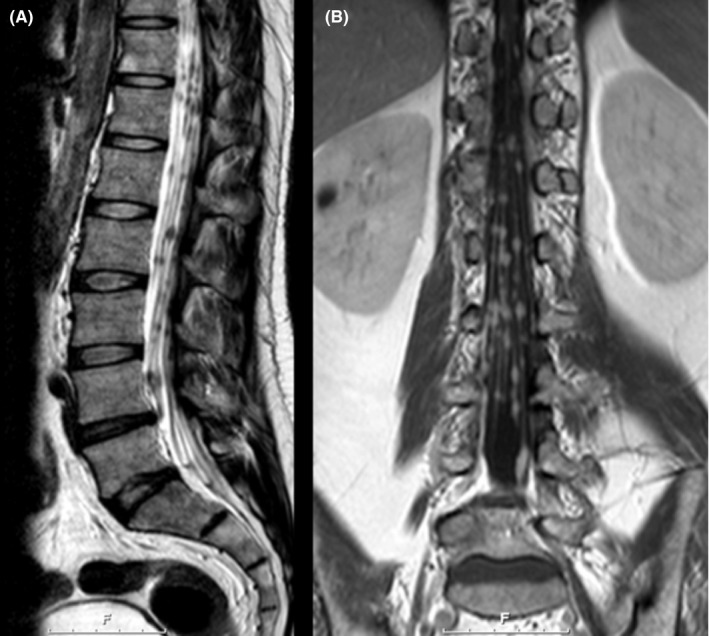
MR Imaging of Lumbar Spine. A, T2‐weighted sagittal images of the lumbar spine (no enhancement); multiple diffuse low signal nodular lesions involving the cauda equina. B, T1‐weighted postgadolinium coronal imaging of the lumbar spine; diffuse nodular enhancement of the cauda equina

**Figure 2:**
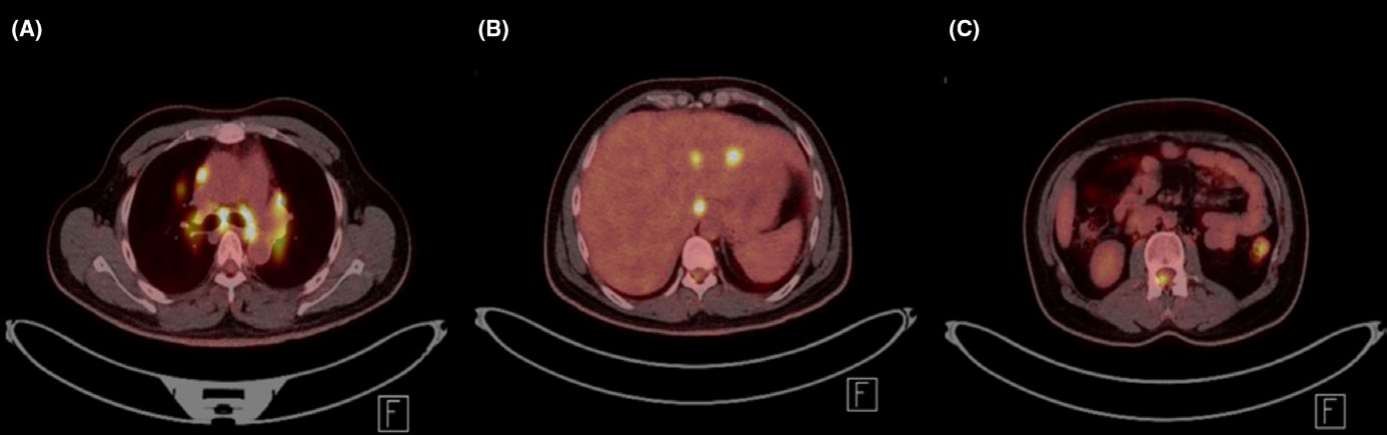
FDG‐PET Imaging. A, Intense metabolic activity associated with pathological mediastinal and bihilar lymphadenopathy. B, Multifocal FDG avid liver lesions. C, Intense focal tracer uptake within the spinal canal compatible with nodular enhancing MR lesions

Magnetic resonance imaging (MRI) of the spine demonstrated diffuse nodular and linear leptomeningeal enhancement along the spinal cord and cauda equina (Figure 1). A plain chest film demonstrated bilateral hilar adenopathy, confirmed by high‐resolution CT chest. ^18^F‐FDG PET scanning demonstrated multifocal, nodular FDG‐avid uptake in the mediastinum, hila, liver, and spinal canal (Figure 2).

Cerebrospinal fluid (CSF) testing revealed a moderate reactive pleocytosis, with a CD4:CD8 ratio of 3.56, protein 3.99 g/L, and glucose 1.7 mmol/L (paired serum glucose 12.6 mmol/L). Cytology was negative for malignant cells. CSF was negative for acid‐fast bacilli, culture, and mycobacterial PCR, and CSF cryptococcal antigen testing was negative. Serum ACE and 24‐hour urinary calcium were normal. Endoscopic bronchial ultrasound of the hilar nodes did not yield adequate tissue for analysis. Ultrasound‐guided percutaneous biopsy of a liver lesion yielded a sample demonstrating multiple granulomas and multinucleate giant cells consistent with sarcoidosis, with staining for acid‐fast bacilli and fungal organisms negative. A diagnosis of systemic sarcoidosis with probable neurosarcoidosis affecting the spinal cord was made.

Following diagnosis, he was treated with pulsed intravenous methylprednisolone, followed by high‐dose oral steroids with slow wean. Azathioprine was started as a steroid‐sparing agent. He responded rapidly with normalization of power and sensation; however, he relapsed repeatedly during steroid wean, and his blood glucose proved difficult to manage while treated with glucocorticoids. He was switched to infliximab with good and sustained response.

## 3 DISCUSSION

Sarcoidosis is a systemic inflammatory condition caused by an exaggerated immune response to an unknown antigen in individuals who are genetically susceptible.^1^ It is characterized by the formation of noncaseating granulomas, typically in the lungs and lymphatics, but can also affect other organs including the skin, eyes, heart, kidneys, and nervous system. The reported prevalence of sarcoidosis is 4.7–64 per 100 000 population, with African American and northern European populations most commonly affected. Peak onset of sarcoidosis is between 25 and 40 years of age.^1^

Symptomatic neurosarcoidosis affects between 3% and 10% of sarcoidosis patients; however, neurological involvement has been reported in 27% of patients at postmortem.^6^ The clinical presentation of neurosarcoidosis is varied; however, cranial neuropathy as a result of either direct granulomatous infiltration, raised intracranial pressure, or basilar aseptic meningitis is the most common manifestation of neurosarcoidosis.^7^ Parenchymal mass lesions, chronic meningitis, neuroendocrine abnormalities, cerebrovascular events, spinal cord, peripheral nerve, and neuromuscular involvement are also well‐recognized manifestations of neurosarcoidosis.^4^ Spinal cord neurosarcoidosis is increasingly recognized, with intramedullary spinal involvement reported in 10%‐25% neurosarcoidosis cases.^2^

Serum biomarkers are associated with sarcoidosis; however, none have been established to be sufficiently sensitive to diagnose neurosarcoidosis. Serum angiotensin‐converting enzyme (ACE) is not sensitive in the diagnosis of neurosarcoidosis, with only 35% cases of neurosarcoidosis found to have elevated serum ACE.^7^ Serum chiothiotridase^8^ and soluble interleukin 2 receptor (sIL‐2R)^9^ have both been reported to be elevated in systemic sarcoidosis, but are not sufficiently sensitive or specific for the diagnosis of neurosarcoidosis.

Cerebrospinal fluid (CSF) examination is indicated in patients with suspected neurosarcoidosis. Mild‐to‐moderate CSF lymphocytosis, with a CD4:CD8 ratio >5, coupled with elevated protein is often seen. Pleocytosis and low glucose may be observed during the acute phase of the disease.^10^ Elevated IgG index and oligoclonal bands are also seen; however, these findings are not specific to neurosarcoidosis. Furthermore, CSF examination is normal in upto 30% of cases of neurosarcoidosis.^4^ Elevated CSF ACE is considered to be have poor sensitivity, and poor specificity.^4^ Elevated sIL‐2R can also be detected in CSF in neurosarcoidosis; however, this is not specific as it may also be elevated in CNS infection.^11^ Cytology and investigations for bacterial, mycobacterial, viral, and fungal infections are important to exclude differential diagnoses.

Gadolinium‐enhanced MRI is the preferred modality of imaging to assess for neurological involvement in sarcoidosis. MRI typically demonstrates high‐intensity lesions on T1 weighting, with postgadolinium enhancement^.^ 12, 13 Characteristic findings include nodular leptomeningeal enhancement, preferential basilar involvement of the brain, and noncontiguous lesions of the spinal cord affecting more than three segments.^4^ Involvement of spinal roots and caudae is also observed in neurosarcoidosis, with nodular leptomeningeal enhancement being characteristic.^12^

The diagnosis of neurosarcoidosis represents a significant challenge as there is a wide differential including infections such as TB, HIV, neurosyphilis, boriella, listeria, and toxoplasmosis; immune‐mediated conditions such as IgG4‐related meningeal disease, CNS vasculitis, granulomatosis with polyangiitis, eosinophilic granulomatosis with polyangiitis, Behçets disease, Sjögrens disease and systemic lupus erythematosus; malignancies including CNS lymphoma, carcinomatous meningitis, leptomeningeal metastasis, and germ cell tumors; and demyelinating conditions such as multiple sclerosis, neuromyelytis optica, and acute demyelinating encephalomyelitis.^4^, ^14^

The modified Zajicek diagnostic criteria^3^, ^4^, ^5^ are outlined in Table 1. Although this diagnostic criterion is widely recognized, it has yet to be systematically validated due to the scarcity of this condition, and the specify and sensitivity of these diagnostic criteria are currently unknown.^14^ Definitive diagnosis of neurosarcoidosis requires positive nervous system histology demonstrating noncaseating epithelioid granuloma; however, this is often not feasible. The diagnosis of probable neurosarcoidosis is typically reliant on histopathological identification of noncaseating granulomas outside the CNS, in this case from a sarcoidosis liver lesion.

**Table 1:** Modified Zajicek criteria for diagnosis of neurosarcoidosis (adapted from Ref. 4)

	Definition
Definitive	Suggestive clinical presentation
	**AND**
	Positive histopathology from neural biopsy
	**AND**
	Exclusion of other disease
Probable	Suggestive clinical presentation
	**AND**
	MRI or CSF evidence of CNS inflammation
	**AND**
	Positive histopathology from extraneural biopsy
	**AND/OR**
	at least two of; FDG‐PET, Gallium Scan, HRCT chest, elevated serum ACE
	**AND**
	exclusion of other disease
Possible	Suggestive clinical presentation
	**AND**
	Exclusion of other disease
	But probable criteria not met

Currently, there are no established guidelines regarding the onset or duration of treatment of sarcoidosis, and there is a paucity of randomized controlled trial data.^1^, ^15^, ^16^ Furthermore, cases of isolated pulmonary sarcoidosis frequently resolve spontaneously within 2 years, and a careful watch and wait strategy is advocated.^1^, ^17^ Indications for immediate treatment of sarcoidosis are generally recognized as functional respiratory impairment or extrapulmonary manifestations including neurological, cardiac, or renal involvement, ocular disease refractory to topical therapy, or symptomatic refractory hypercalcaemia^1^, ^15^, ^16^, ^18^

Immediate treatment for patients with confirmed systemic sarcoidosis with evidence of CNS involvement has been advocated,^4^, ^15^, ^16^, ^18^ as unlike pulmonary sarcoidosis spontaneous resolution of neurosarcoidosis is rare, and there is significant risk of morbidity and mortality.^19^ Treatment with corticosteroids, typically 1 mg/kg/d is first line for neurosarcoidosis. For patients presenting with severe disabling disease such as brain or spinal cord lesions, leptomeningeal involvement or hydrocephalus, aggressive management with pulsed IV methylprednisolone followed by prolonged corticosteroid wean over six to twelve months is recommended.^4^, ^18^ Treatment with disease‐modifying antisarcoidosis drugs (DMASDs) such as methotrexate, azathioprine, or cyclophosphamide has been reported for patient with disease refractory to glucocorticoid monotherapy, or when the side effect profile of glucocorticoids is prohibitory.^4^, ^18^, ^20^ For patients with disease refractory to combination corticosteroid/DMASD therapy, successful treatment with the antitumor necrosis factor‐α monoclonal antibody infliximab has been reported.^4^, ^15^, ^16^, ^18^, ^21^ Furthermore, first‐line treatment with infliximab has also been advocated by some experts for patients presenting with severe disabling disease which requires a rapid response to avoid permanent disability.^4^, ^18^ Neurosarcoidosis is a rare disease entity and referral to a specialist center is recommended.

A recent meta‐analysis of outcomes of neurosarcoidosis patients reported complete or partial remission in 59%, stable disease in 24%, and deterioration in 6% of treated patients. Overall mortality is significant with 42 deaths reported in 826 patients (5%, CI 95 4‐7%).^7^

## AUTHORSHIP

CO: Medical Registrar; responsible for inpatient care, patient consent, synthesis of case report, and primary author of manuscript, corresponding author. FJ: Consultant neuroradiologist; responsible for report of neuroimaging, critical revising, and final approval. AB: Consultant neurologist; senior physician responsible for patient care, critical revising, and final approval.

## CONFLICT OF INTEREST

None declared.
